# Biosynthesis and Characterization of Silver Nanoparticles by *Aspergillus* Species

**DOI:** 10.1155/2016/5435397

**Published:** 2016-08-29

**Authors:** Kamiar Zomorodian, Seyedmohammad Pourshahid, Arman Sadatsharifi, Pouyan Mehryar, Keyvan Pakshir, Mohammad Javad Rahimi, Ali Arabi Monfared

**Affiliations:** ^1^Basic Sciences in Infectious Diseases Research Center, Shiraz University of Medical Sciences, Shiraz, Iran; ^2^Department of Medical Mycology and Parasitology, School of Medicine, Shiraz University of Medical Sciences, Shiraz, Iran; ^3^Institute of Design, Robotics and Optimisation, School of Mechanical Engineering, University of Leeds, Leeds, UK; ^4^Microbiology Group, Research Area Biotechnology and Microbiology, Institute of Chemical Engineering, Vienna University of Technology, Vienna, Austria

## Abstract

Currently, researchers turn to natural processes such as using biological microorganisms in order to develop reliable and ecofriendly methods for the synthesis of metallic nanoparticles. In this study, we have investigated extracellular biosynthesis of silver nanoparticles using four* Aspergillus* species including* A. fumigatus*,* A. clavatus*,* A. niger, *and* A. flavus*. We have also analyzed nitrate reductase activity in the studied species in order to determine the probable role of this enzyme in the biosynthesis of silver nanoparticles. The formation of silver nanoparticles in the cell filtrates was confirmed by the passage of laser light, change in the color of cell filtrates, absorption peak at 430 nm in UV-Vis spectra, and atomic force microscopy (AFM). There was a logical relationship between the efficiencies of studied* Aspergillus* species in the production of silver nanoparticles and their nitrate reductase activity.* A. fumigatus* as the most efficient species showed the highest nitrate reductase activity among the studied species while* A. flavus* exhibited the lowest capacity in the biosynthesis of silver nanoparticles which was in accord with its low nitrate reductase activity. The present study showed that* Aspergillus *species had potential for the biosynthesis of silver nanoparticles depending on their nitrate reductase activity.

## 1. Introduction

Nanotechnology deals with the synthesis and stabilization of matters at the nanoscale ranging from 1 to 100 nm [1]. Nanomaterials have novel and size-related physicochemical properties which are significantly different from their macroscopic properties [2]. Nanoparticles as the cornerstone of nanomaterials are the starting points for preparing many nanostructures so their synthesis is considered as an important part of the research attempts in nanoscience and nanoengineering [3]. Sliver nanoparticles are one of the most widely used nanoparticles [4]. In recent years, silver nanoparticles have found enormous applications in various fields such as biolabeling, sensors, antimicrobial agents, filters, microelectronics, and catalysis, because of their specific physiochemical and biological properties [5–10]. These nanoparticles have no toxic effects on humans but have inhibitory effects on the growth of bacteria, virus, and other eukaryotic microorganisms [11]. In addition to their distinctive properties, their production cost is relatively low [12].

Traditionally, physical and chemical methods have been utilized for the synthesis of nanoparticles [13, 14]. Basically, the physical methods have low yields and the chemical methods have harmful effects on the environment due to use of toxic solvents and the generation of hazardous by-products [15]. Currently, scientists focus on biosynthesis of nanoparticles using bacteria [16], fungus [8], and plants [17]. These biogenic processes are of low cost and high yield, safe, and ecofriendly in comparison with the physical and chemical synthetic procedures [3].

Fungi secrete large amounts of enzymes and are easy growing on every medium so they are considered as a proper choice for the biosynthesis of nanoparticles [18, 19]. Many studies have been done so far using various species of fungi for the biosynthesis of sliver nanoparticles such as* Aspergillus* [8, 20, 21],* Fusarium* [22, 23],* Penicillium* [24, 25],* Trichoderma* [26, 27], and* Cladosporium* [28]. Among minerals, fungi require nitrogen in the largest amounts, so nitrogen can be considered as the limiting factor for their growth can be accounted. Unlike bacteria, fungi cannot fix atmospheric nitrogen, but they are able to use many other forms of nitrogen like amino acids, ammonium, and nitrate. Several fungi can convert nitrate as sole nitrogen source to ammonium by the enzymes nitrate reductase and nitrite reductase [29].

The precise reaction mechanism leading to the biosynthesis of sliver nanoparticles is yet to be clarified. Previous studies proposed the probable role of the reduced form of nicotinamide adenine dinucleotide (NADH) and NADH-dependent nitrate reductase in the reduction of silver ion to metallic silver [18, 30]. In the present study, we have investigated the extracellular biosynthesis of sliver nanoparticles using four* Aspergillus* species including* A. fumigatus*,* A. clavatus*,* A. niger, *and* A. flavus*. In order to determine the probable role of nitrate reductase in the formation of sliver nanoparticles, we have analyzed the relationship between the quality and quantity of biosynthesized sliver nanoparticles by the studied* Aspergillus* species and their nitrate reductase activity.

## 2. Materials and Methods

### 2.1. Microorganisms

The reference species of following fungi and bacteria were obtained from the American Type Culture Collection (ATCC), the Centraalbureau voor Schimmelcultures (CBS), and Teikyo University Institute of Medical Mycology (TIMM):* Aspergillus flavus* (ATCC® 64025*™*),* A. fumigatus* (ATCC® 14110*™*),* A. clavatus* (CBS® 514.65*™*),* A. niger* (TIMM® 0113*™*),* Pseudomonas aeruginosa* (ATCC® 27853*™*), and* Enterococcus faecalis* (ATCC® 11700*™*). Fungi were used for the biosynthesis of silver nanoparticles and bacteria were used as a positive control in nitrate reduction test.

Fungi were subcultured in Sabouraud Dextrose Agar (Merck, Germany) and incubated at 35°C for 24 hours. In order to prevent the bacterial growth, 50 mg/L chloramphenicol (Sigma-Aldrich, Germany) was added to the cultures. Bacteria species were subcultured in Brain Heart Infusion Agar (Merck, Germany) and were incubated at 37°C for 24 hours.

### 2.2. Nitrate Reduction Test

Equal biomass of microorganisms was cultured in appropriate media and used for the test. Nitrate reduction was determined by using nitrate reduction test kit (Fluka 73426, Sigma-Aldrich). Fungi were categorized into three different groups including high (+++), intermediate (++), and low (+) nitrate reductase enzyme activity.

### 2.3. Biomass Production

To prepare biomass, fungi were grown aerobically in liquid media containing (g/L) KH_2_PO_4_, 7.0; K_2_HPO_4_, 2.0; MgSO_4_·7H_2_O, 0.1; (NH_4_)_2_SO_4_, 1.0; yeast extract, 0.6; and glucose, 10.0. The flasks were inoculated and incubated on orbital shaker at 150 rpm at 25°C. The biomass was harvested after 72 hours of growth by filtering through a paper sieve, followed by substantial washing with distilled deionized water in order to remove any medium component from the biomass.

### 2.4. Biosynthesis of Silver Nanoparticles

Typically 20 g of fresh biomass was added to 200 mL of Milli-Q deionized water for 48 hours at 25°C in an Erlenmeyer flask and agitated on orbital shaker at 150 rpm. After incubation, the cell filtrate was obtained by sieving the content through Whatman filter paper no. 1. For synthesis of silver nanoparticles, a precise weighed quantity of AgNO_3_ was added to the cell filtrate of each of the fungi in an Erlenmeyer flask to produce an overall silver ion (Ag^+^) concentration of 1 mM and agitated at 25°C in dark. A control flask (without the silver ion) was also included in each experiment along with the test flask.

### 2.5. Characterization of Silver Nanoparticles

The preliminary characterization of sliver nanoparticles was done by visual observation of color change in the cell filtrate and transmission of laser light. The UV-Vis spectroscopy measurements were performed by using a UV-Vis spectrophotometer (Spectronic 20, Arthur H. Thomas Co., USA) in the range of 300–500 nm with the resolution of 10 nm after 48 hrs. The particle size distribution of sliver nanoparticles was determined by using Zeta-Sizer Nano ZS (Malvern Instruments, Southborough, UK). The morphology of sliver nanoparticles was studied by atomic force microscopy (AFM) (NanoWizard II, JPK Instruments, Germany). A thin layer of the sample was prepared by dropping 100 *μ*L of the sample on a glass slide and was allowed to dry for 5 minute. The AFM images were then taken with silicon cantilevers (CSC17, MikroMasch, Estonia) in contact mode. The AFM images were processed using JPK data processing software (JPK Instruments, Germany).

### 2.6. Statistical Analysis

Data were presented as the mean ± standard deviation (SD). Statistical analysis was performed by one-way ANOVA test using the SPSS version 19 software package (SPSS, Chicago, IL, USA). A *P* value < 0.05 was considered significant.

## 3. Results and Discussion

**Figure 1:**
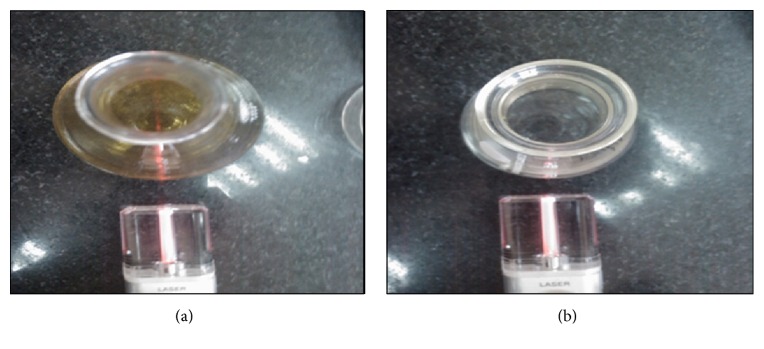
Characterization of sliver nanoparticles by color formation in the cell filtrate and laser light. (a) The sample of* A. fumigatus* cell filtrate containing sliver nanoparticles (dark brown color) and (b) the sample of its control.

After incubation of the fungal cell filtrates with silver ions and maintenance in the dark, the cell filtrates showed a gradual change in color towards brown, which was in accord with the results of previous studies [8, 18, 24]. The color of the cell filtrates changed to intense brown after 48 h of incubation. This indicated the formation of sliver nanoparticles in the medium which was mainly due to the excitation of surface plasmon vibrations in the nanoparticles [31]. Controls (without silver ion) exhibited no change in color of the cell filtrate in the same condition of incubation. The biosynthesis of sliver nanoparticles was detected by transmission of laser light through the cell filtrate as well.  Figure 1(a) shows the color formation and the passage of laser light through the sample of* A. fumigatus *cell filtrate containing sliver nanoparticles in comparison with its control (Figure 1(b)).

**Figure 2:**
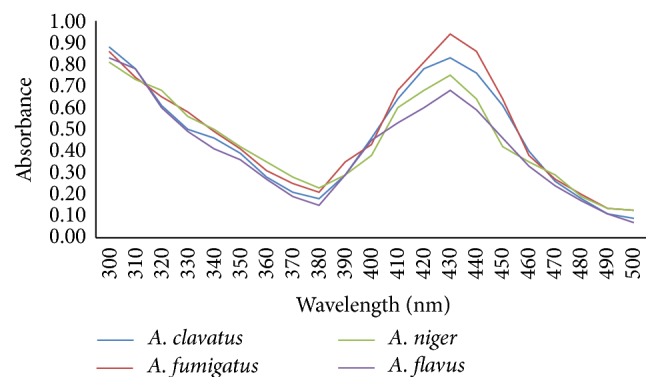
UV-Vis absorption spectra of silver nanoparticles produced by* A. fumigatus*,* A. clavatus, A. niger,* and* A. flavus* after 48 h of incubation.

The UV-Vis absorption spectra recorded from the studied* Aspergillus* species reaction vessel after 48 h of incubation are plotted in Figure 2. The absorption spectra indicated a peak at 430 nm for all* Aspergillus* species, attributable to the surface plasmon resonance band of sliver nanoparticles [32]. At this wavelength, the maximum intensity of absorption belonged to sliver nanoparticles synthesized by* A. fumigatus* and the intensity of absorption decreased in* A. clavatus*,* A. niger,* and* A. flavus*, respectively. The intensity of the peaks is related to the amount of produced sliver nanoparticles so we can conclude that* A. fumigatus* produced greater amount of sliver nanoparticles compared with the three other fungi. The amount of biosynthesized sliver nanoparticles reduced in* A. clavatus*,* A. niger,* and* A. flavus*, respectively.

The results of particle size analysis of biosynthesized sliver nanoparticles in each of the samples are summarized in Table 1. There was a significant difference between the particle sizes of sliver nanoparticles produced by studied fungi (*P* value < 0.01).* A. fumigatus* produced smaller sliver nanoparticles (mean = 49.00) with higher monodispersity (SD = 19.64) in comparison with the other species. The sliver nanoparticles biosynthesized by* A. clavatus*,* A. niger,* and* A. flavus* became larger and more polydisperse in the order mentioned.

**Figure 3:**
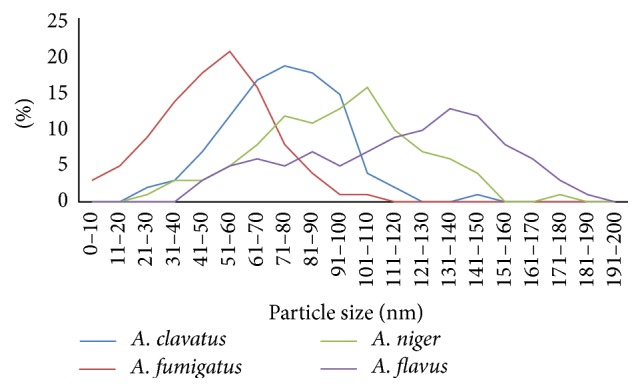
Particle size distribution curves of silver nanoparticles synthesized by* A*.* fumigatus, A*.* clavatus*,* A*.* niger,* and* A*.* flavus*.

The particle size distribution curves of sliver nanoparticles confirm the results of particle size analysis completely (Figure 3). In these curves, sharp peaks show a narrow distribution of sliver nanoparticles size while broad peaks indicate a wide distribution.

**Figure 4:**
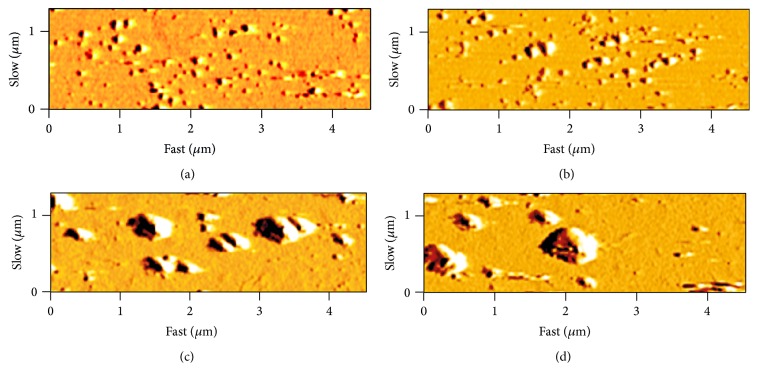
AFM images of biosynthesized silver nanoparticles by (a)* A. fumigatus*, (b)* A. clavatus*, (c)* A*.* niger,* and (d)* A*.* flavus*.

In the study of AFM, the morphology of synthesized sliver nanoparticles was found to be highly variable, most of them presented in spherical shape (Figure 4). As shown in this figure, the silver nanoparticles produced by* A. fumigates* were smaller in size and more monodisperse in comparison with those produced by the other examined species.

The reaction mechanism leading to the formation of silver nanoparticles is not definitely realized yet. The previous studies showed the secretion of some proteins into the medium by the fungi which might play important role in the reduction of silver ions in the form of nanoparticles and the stabilization of them [8, 18, 30]. Bhainsa and D'Souza reported the UV-Vis spectrum study on silver nanoparticles produced by the fungus,* A. fumigatus*. They found two absorbance peaks in the UV range corresponding to 220 nm (may be due to absorption by amide bond) and 280 nm (may be attributed to the tryptophan and tyrosine residues in the proteins) which indicated the secretion of some proteinic components into the medium by* A. fumigatus* [8]. In another study, Ahmad et al. investigated the extracellular biosynthesis of silver nanoparticles by* Fusarium oxysporum*. They reported that* F. oxysporum* secreted NADH-dependent reductase which was probably responsible for the reduction of silver ions and the formation of silver nanoparticles [18].

In this study, we determined nitrate reductase activity in the studied* Aspergillus* species. In nitrate reduction test,* A. fumigatus* exhibited a high nitrate reductase activity.* A. clavatus* and* A. niger* had an intermediate enzymatic activity and nitrate reductase activity was low in* A. flavus*.

Our findings showed a reasonable relationship between nitrate reductase activity and the efficiency of studied* Aspergillus* species in the production of silver nanoparticles.* A. fumigatus* as the most efficient species had the highest nitrate reductase activity among the studied fungi. It produced greater amount of silver nanoparticles with smaller size and higher monodispersity in comparison with other species. On the other hand,* A. flavus* exhibited the lowest capacity in the production of silver nanoparticles which was in agreement with its low nitrate reductase activity. Hence, the difference in the biosynthesis of silver nanoparticles between the studied* Aspergillus* species might be related to their different ability in the production of nitrate reductase enzyme. Our findings indicate the probable role of this enzyme in the formation of silver nanoparticles.

## 4. Conclusions

In the present study,* A. fumigatus*,* A. clavatus*,* A. niger,* and* A. flavus* have shown potential for extracellular biosynthesis of silver nanoparticles, as confirmed by UV-Vis spectroscopy and AFM. The biosynthesized silver nanoparticles showed a characteristic absorption peak at 430 nm in UV-Vis spectra. In the study of AFM, majority of the silver nanoparticles were spherical in shape. Apparently, the capacity of studied* Aspergillus* species in the production of silver nanoparticles is depending on their nitrate reductase activity. In nitrate reduction test, the highest enzymatic activity was observed in* A. fumigatus* which was the most efficient species in the production of silver nanoparticles in terms of both quantity and quality compared with other studied species. However, chemical composition and concentration of the secondary metabolites secreted by the fungi might also have a role in such differences. Therefore, further studies are still needed to understand the precise molecular mechanism leading to the formation of silver nanoparticles by biological methods in order to have a better control over the size and polydispersity of these nanoparticles.

## Figures and Tables

**Table 1:** Particle size analysis data of silver nanoparticles.

Fungus	Particle size (nm)^a^ (mean ± SD)	Size range (nm)
*Aspergillus fumigatus*	49.00 ± 19.64	5–95
*Aspergillus clavatus*	74.20 ± 21.20	25–145
*Aspergillus niger*	93.30 ± 28.39	25–175
*Aspergillus flavus *	117.00 ± 35.50	45–185

^a^Significant difference between the particle sizes of silver nanoparticles based on one-way ANOVA test (*P* value < 0.01).
